# The Effect of Holographic Heart Models and Mixed Reality for Anatomy Learning in Congenital Heart Disease: An Exploratory Study

**DOI:** 10.1007/s10916-023-01959-8

**Published:** 2023-05-17

**Authors:** Angelo Fabio d’Aiello, Federico Cabitza, Chiara Natali, Sophia Viganò, Paolo Ferrero, Ludovica Bognoni, Giulia Pasqualin, Alessandro Giamberti, Massimo Chessa

**Affiliations:** 1 ACHD UNIT, Pediatric and Adult Congenital Heart Centre, IRCCS Policlinico San Donato, Piazza Edmondo Malan 2, San Donato Milanese, 20097 Italy; 2 European Reference Network for Rare and Low Prevalence Complex Disease of the Heart (ERN GUARD-Heart), Milan, Italy; 3 Department of Informatics, Systems and Communication, University of Milano-Bicocca, viale Sarca 336, Milano, 20126 Italy; 4 IRCCS Istituto Ortopedico Galeazzi, Via Cristina Belgioioso 173, Milano, 20157 Italy; 5 School of Medicine and Surgery, Vita e Salute San Raffaele University, Via Olgettina 58, Milano, 20132 Italy

**Keywords:** Augmented reality, Mixed reality, Congenital heart disease, Holographic images, Medical education, HoloLens

## Abstract

In this paper, we present an exploratory study on the potential impact of holographic heart models and mixed reality technology on medical training, and in particular in teaching complex Congenital Heart Diseases (CHD) to medical students. Fifty-nine medical students were randomly allocated into three groups. Each participant in each group received a 30-minute lecture on a CHD condition interpretation and transcatheter treatment with different instructional tools. The participants of the first group attended a lecture in which traditional slides were projected onto a flat screen (group “regular slideware”, RS). The second group was shown slides incorporating videos of holographic anatomical models (group “holographic videos”, HV). Finally, those in the third group wore immersive, head-mounted devices (HMD) to interact directly with holographic anatomical models (group “mixed reality”, MR). At the end of the lecture, the members of each group were asked to fill in a multiple-choice questionnaire aimed at evaluating their topic proficiency, as a proxy to evaluate the effectiveness of the training session (in terms of acquired notions); participants from group MR were also asked to fill in a questionnaire regarding the recommendability and usability of the MS Hololens HMDs, as a proxy of satisfaction regarding its use experience (UX). The findings show promising results for usability and user acceptance.

## Introduction

This paper focuses on the role of Mixed-Reality (MR) technology in the learning of Congenital Heart Diseases (CHD) and related treatments, such as the sinus venosus atrial septal defect (SV-ASD), the superior vena cava type with partial anomalous pulmonary venous return (PAPVR) and its transcatheter treatment. To this aim, we will briefly outline the current state of the art in virtual 3D models and objects, which are typical in MR for medical training and education, and we will report on a small-scale application of a commercial MR technology during a lecture on CHD diagnosis and treatment. The aim of our user study is to assess whether *immersive* 3D models enable better knowledge acquisition than 3D models displayed on a 2D screen, and than traditional teaching materials. The discussion will cover the potential benefits and challenges of MR technology in the field of medical training, and we will propose some future directions for research and development.

The teaching of CHD greatly relies on the use of two-dimensional images, often in the form of simple diagrams; however, these images cannot convey depth perception and leave a lot of the understanding to individual spatial imagination, ultimately complicating the learning process [1, 2]. To address these limitations, physicians have developed new techniques that allow for the three-dimensional visualization of CHDs, including MR technology.

MR technology (sometimes also called holographic technology in the educational field [3]) combines elements of both virtual and physical worlds to be experienced via headsets. While similar to Virtual Reality (VR) in its real-time simulation of visual sensation and in tracking the user’s position and movements (usually through head-mounted display), MR does not fully immerse users in a virtual 3D space but rather projects virtual elements into a real-world environment. This link between real and virtual environments should help in reducing adverse health effects, such as cybersickness [4], blurred vision, and disorientation, which are often experienced in virtual reality applications that use head-mounted displays [5]. MR technologies can provide immersive, interactive learning experiences for medical students and practitioners to help them develop the skills and knowledge they need [6], and therefore they have the potential to revolutionize undergraduate scientific education [7, 8] as well as medical training [9–11]. An example of this is an MR system called ARS-CADPT, which was developed for coronary artery diagnosis and preoperative planning [12] and offered real-time, user-friendly human-computer interaction via gesture recognition. CHD training is a suitable field for testing the effectiveness of holographic technology since the pathophysiology of CHDs is strictly related to the underlying anatomical anomaly; therefore, the understanding of the complex three-dimensional anatomy of these conditions is essential to assess pathophysiological consequences and guide treatment decisions. The benefit of 3D holograms compared to printed images has been shown to significantly improve anatomical knowledge and understanding [13], due to a suspected reduction in cognitive load [14, 15]

Several studies on 3D holograms for surgical training for CHD showed increased student motivation, strengthened learning efficiency and better communication mechanisms between teachers and students. [16] The HoloLens device outperformed traditional approaches in the teaching of cardiovascular disease on several pedagogy measurements, including student experience, satisfaction, problem-solving skills, and clinical reasoning. [17] Different studies in the fields of neuroanatomy and neurophysiology [18, 19] point to less conclusive results: in one case, students who studied from a traditional projector-based Microsoft PowerPoint significantly outperformed the HoloLens group in an anatomy identification test and brain physiology question test, while reporting higher mean test anxiety scores; however, the HoloLens group showed a significantly higher preference to the learning experience compared to the PPT group based on a systems self-efficacy questionnaire. [19] In another comparison between lessons delivered through HoloLens and through a handheld tablet device, [18] no significant differences were found in test scores between the two methods, while reporting a significant increase in dizziness with the use of HoloLens. Still, even when no differences emerged in formative value, other researches showed that students expressed the desire to be trained both in traditional and innovative technology for pre-operative planning [15] due to its engaging nature [20], also pointing to its reduced cognitive load and effort, shorter response time and more positive emotions. [15] In our study, we will focus on the pedagogical potential of Mixed Reality experiences via the Microsoft HoloLens device, testing its effectiveness (measured as increased participant knowledge) and the user experience of students in comparison to traditional means (regular slides) and 2D holographic videos.

## Materials and method

### Participants and models

For this study, we enrolled fifty-nine medical students from the Vita-Salute San Raffaele University, Milan, Italy during their third academic year, just after the teaching of the course on general cardiology. During the lessons, two models were used: one to show the anatomy, and one to show the interventional treatment. Both models were patient-specific and were derived from CT (Computerized Tomography) dataset.

### Instructional tools

**Fig. 1:**
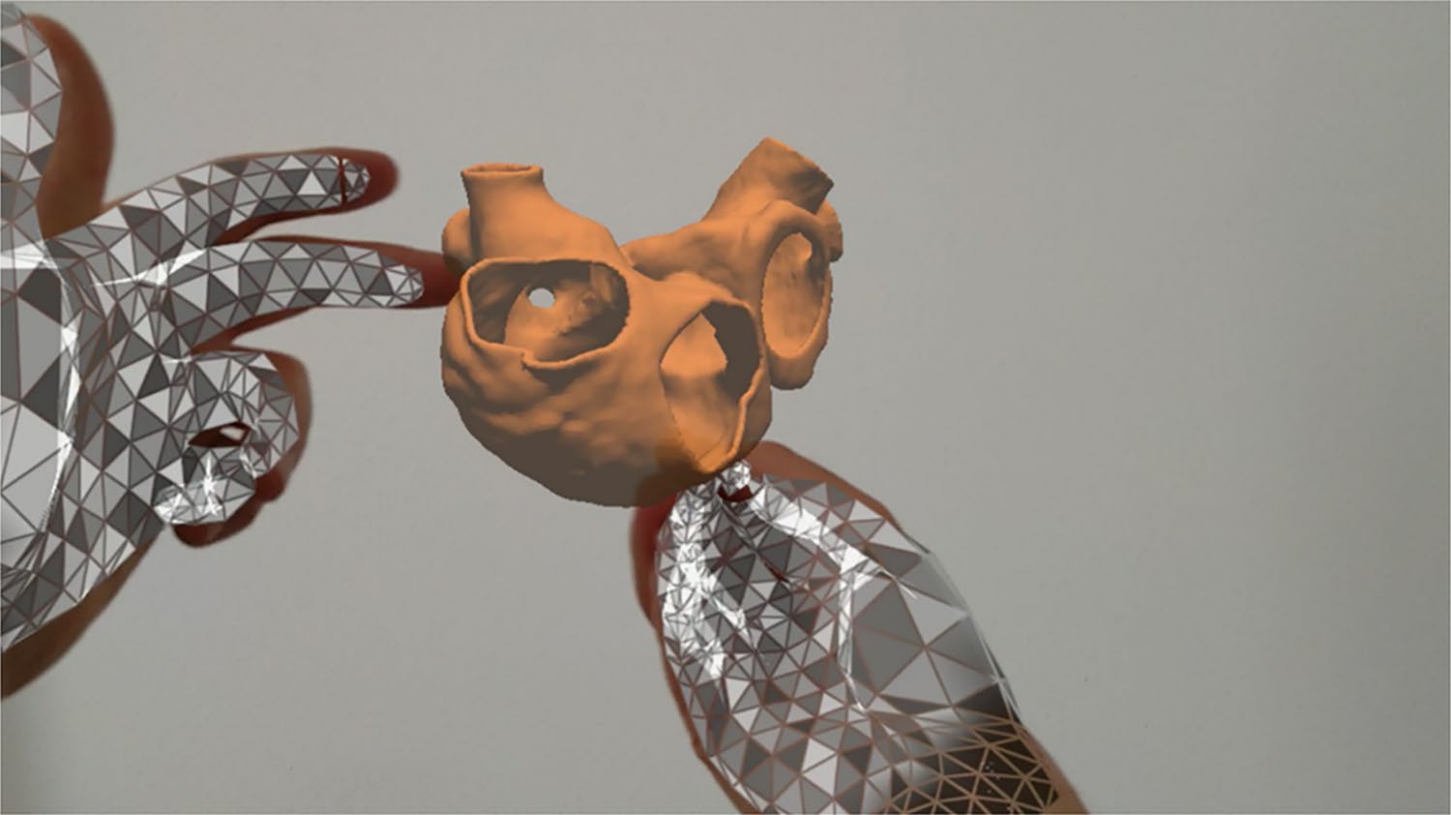
First-person view of the user interaction with the anatomical 3D model using the R&D version of ARTICOR. The model (light brown) can be visualized in the 3D space and hand tracking (meshed silver gloves) can be used to interact with the model itself

The hardware used for this study was Microsoft HoloLens 2.0 (Microsoft Corp., Redmond, WA, USA) running on Windows Holographic Operative system. The device can perform hand, head tracking and spatial mapping of the room, which together allow for a holographic visualization and intuitive interaction with the virtual objects while allowing for complete awareness of one’s actual surroundings. On the headsets an R&D version of the software platform ARTICOR^®^(developed by Artiness srl, Milano, Italy) was installed, for the visualization and interaction with holographic 3D images and models in the medical field (Fig. 1).

**Fig. 2:**
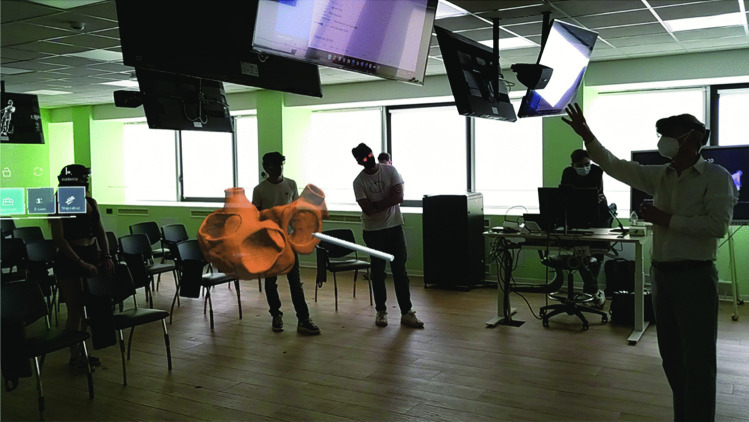
First-person view of the user during a collaborative session

**Fig. 3:**
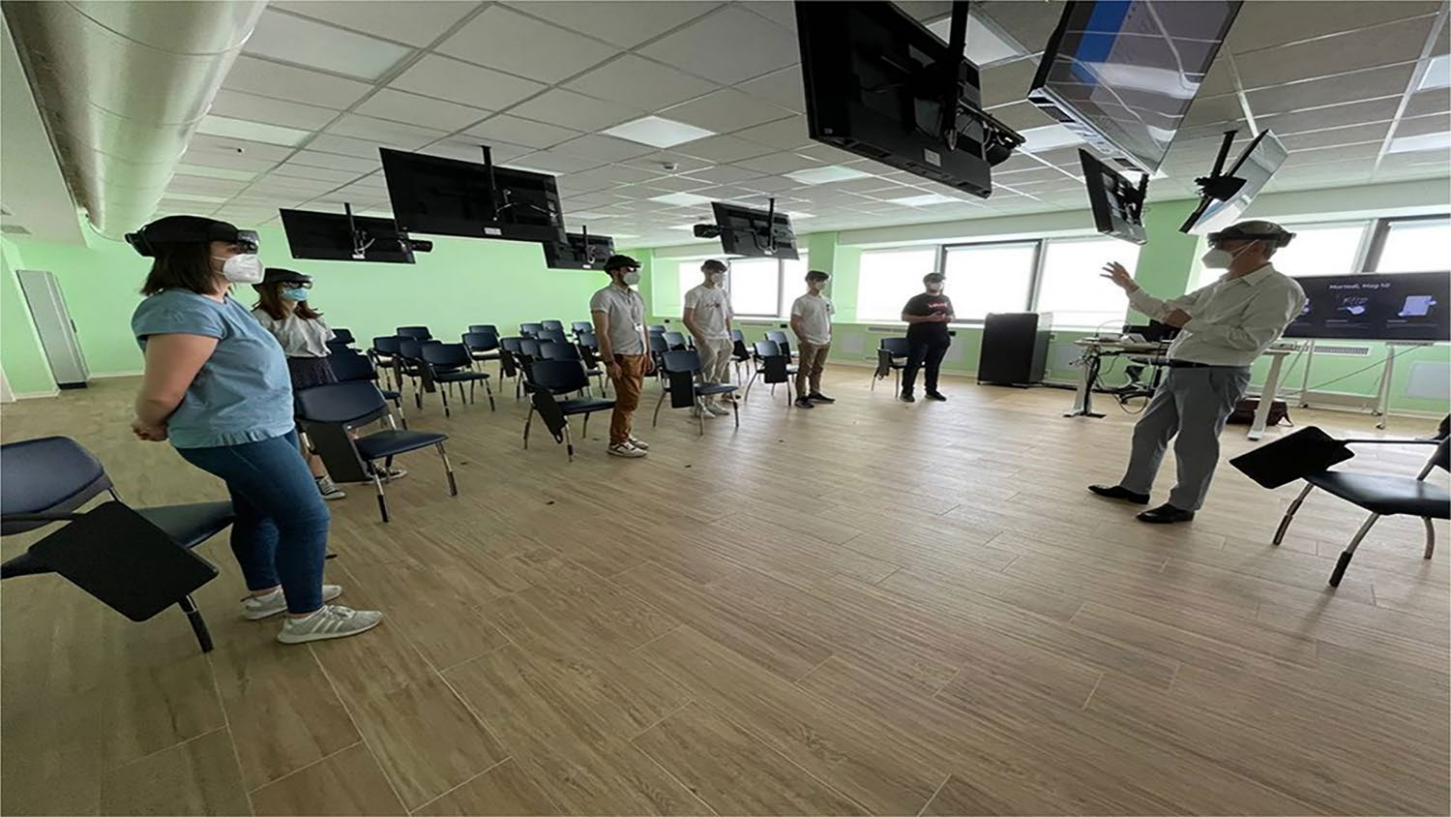
Collaborative session between six students and the instructor

The software platform allowed all HoloLens devices, i.e., all participants (the teacher and the students), to be virtually located in the same collaborative session with the relative position and orientation of the 3D models synced between all of them (Figs. 2 and 3).

### Teaching procedure

The method and instructional tool used to deliver the lesson were the experimental independent variable. The medical students were randomly allocated into three groups (group RS, group HV, and group MR, see Fig. 3) of 20, 20 and 19 people respectively. The lessons were organized so that the three groups had the same amount of time available. In detail, each participant in each group received a 30-minute recorded lecture on SV-ASD superior vena cava type with PAPVR, and its transcatheter treatment. The only difference was in the use of the images between groups, as the lectures were enriched with distinct instructional tools: the first group attended a traditional lecture with slides projected onto a flat screen (group “regular slideware”, RS). The second group was shown slides incorporating videos of holographic anatomical models of SV-ASD with PAPVR anatomy, and transcatheter intervention planning, both displayed as a non-interactive 2D video on a on a traditional projector screen (group “holographic videos”, HV). The same holographic 3D model was also used for the “mixed reality” group. The MR group interacted with holographic images of the CHD and SV-ASD anatomy, procedural planning and procedural results wearing an immersive device during 3 collaborative sessions of 6 students in a large room: during each lecture, 8 MR devices (HoloLens) were available: all students in the MR group and the professor wore Hololens and could interact with holographic models. Since participants in the RS group were shown anatomic 2D images displayed on a traditional projector screen, their group can be considered the control (or no intervention) group.

### Assessment and questionnaires

At the end of the lecture, each group completed a multiple-choice questionnaire to evaluate their topic proficiency, as a proxy to evaluate the effectiveness of the training session (in terms of acquired notions); participants from group MR were also asked to fill in a questionnaire regarding the recommendability and usability of the MS HoloLens HMDs, as a proxy of satisfaction regarding the use experience (UX) of that instructional tool. The proficiency test consisted of ten multiple-choice questions: five questions on epidemiology, anatomy, and pathophysiology of the disease, and five questions aimed at assessing the understanding of the anatomical relationships between the right pulmonary veins, the superior vena cava (SVC) and sinus venosus ASD and their implication in the transcatheter treatment of the defect (see Tables 2 and 3 in the Appendix). Correct answers in the proficiency test were worth one point and wrong answers were worth zero points, with accuracy defined as the average score across all ten questions. On the other hand, the UX questionnaire encompassed 9 closed-ended items (see Table 4 in Appendix). The first item was closely inspired by the Net Promoter Score [21]. Responses to this item higher than 8 (i.e., either 9 s or 10 s) are associated with so called “promoters” respondents (in regard to the solution/product under examination); scores encompassing either 7 s or 8 s are associated to so called “passive” users; respondents who scores 6 or less are labeled as “Detractors” and hence to unsatisfied users. More precisely, the Net Promoter Score (NPS) is calculated by subtracting the percentage of detractors from the percentage of promoters, and therefore it ranges from -100 to 100. The other items of the UX test were aimed to assess the responder’s perceptions of the usefulness and usability of the immersive devices for learning purposes. All items were rendered in terms of a Likert scale with six options, to avoid central tendency bias [22], ranging from totally disagree (1) to totally agree (6).

### Statistical analysis and results

Inference statistical analysis was conducted at a 95% confidence level.

**Fig. 4:**
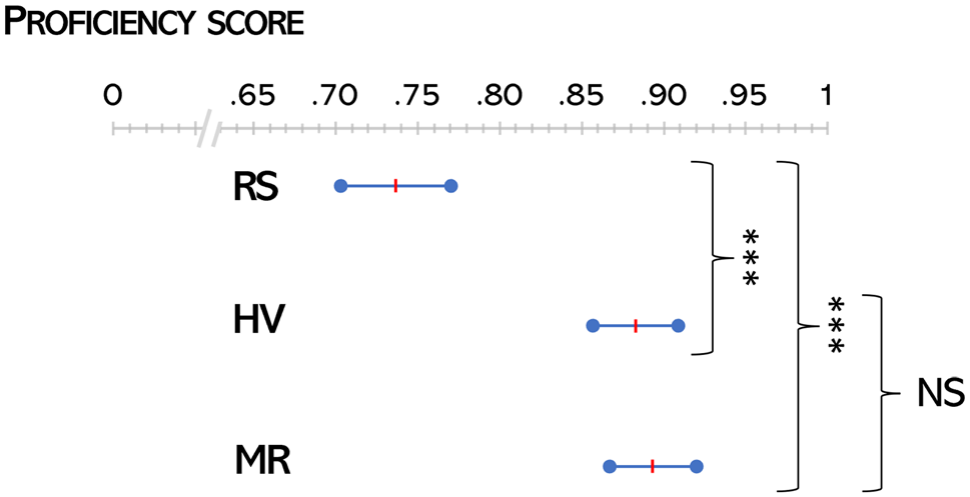
Average Student performance (in terms of accuracy) in the proficiency test, for group RS, HV and MR. Red lines indicate average scores, while segmented indicate the corresponding 95% confidence intervals. Asterisks indicate highly significant difference in average accuracy

All students filled in the proficiency questionnaire, with no missing items. Their performance, summarized in Table 1, was evaluated in terms of accuracy, which is defined as the complement 1 of the error rate. In turn, the error rate is the number of wrong answers out of all the questions in the test: therefore, the higher the accuracy, the better. The average accuracy across all students was .83 (SD=.14, min=.6, max=1.0). The average accuracy in the groups RS, HV, and MR was, respectively,.74 (.14, 1, .6),.88 (.13, 1, .6), and.89 (.08, 1,. 7), see Fig. 4. The difference in performance between group RS and HV and between group RS and MR, assessed through a two-proportion Z test, was statistically significant (Z=$-$3.6, p-value<0.001 and Z=3.8, p-value<0.001, respectively), unlike the difference between group HV and MR (Z=-.03, p-value=.76). Effect sizes (i.e. the intensity of the difference between two grups) were medium according to common interpretation guidelines [23]: for group HV we observed an effect size of .38 with respect to control group (RS); for group MR we reported an effect size of .41. The difference in effect size between group HV and MR was small (d=.05).

**Table 1:** Results of the proficiency test, group by test group (RS, HV and MR) and by type of items (All, knowledge & Anatomy,Treatment). We report relative frequencies of correct responses, average accuracy of each group, and 95% confidence intervals of the averabe accuracy

Accuracy						
All items	group	correct resp	total resp	Avrg acc	[inf	sup]
	RS	155	210	0.74	0.71	0.77
	HV	168	190	0.88	0.86	0.91
	MR	161	180	0.89	0.87	0.92
	Total	484	580	0.83	0.82	0.85
Only knowledge and Anatomy items						
	RS	114	126	0.90	0.88	0.93
	HV	104	114	0.91	0.89	0.94
	MR	101	108	0.94	0.91	0.96
	Total	319	348	0.92	0.90	0.93
Only treatment items						
	RS	41	84	0.49	0.43	0.54
	HV	64	76	0.84	0.80	0.88
	MR	60	72	0.83	0.79	0.88
	Total	165	232	0.71	0.68	0.74

**Fig. 5:**
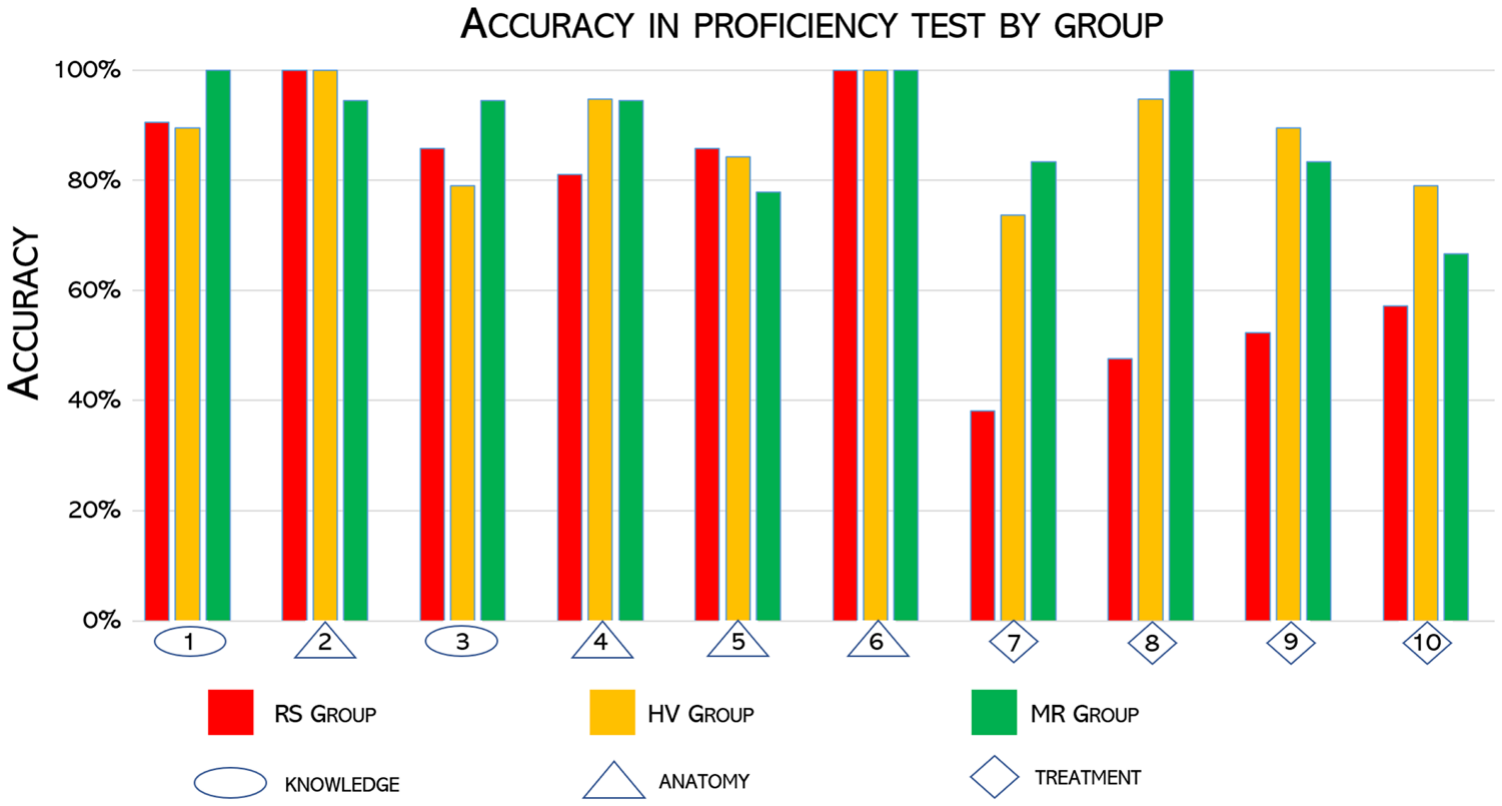
Barplot of the student performance (in terms of accuracy, the higher the better) in the proficiency test, grouped for item (from 1 to 10) and test group (RS, HV and MR). See Appendix Tables 2 and 3 for the item text

However, performance across different items was not uniform: Students from group RS performed much worse than group HV and MR in the last four questions, which were those regarding percutaneous treatment with stenting (see Fig. 5 and Table 3 in the Appendix for the item text).

**Fig. 6:**
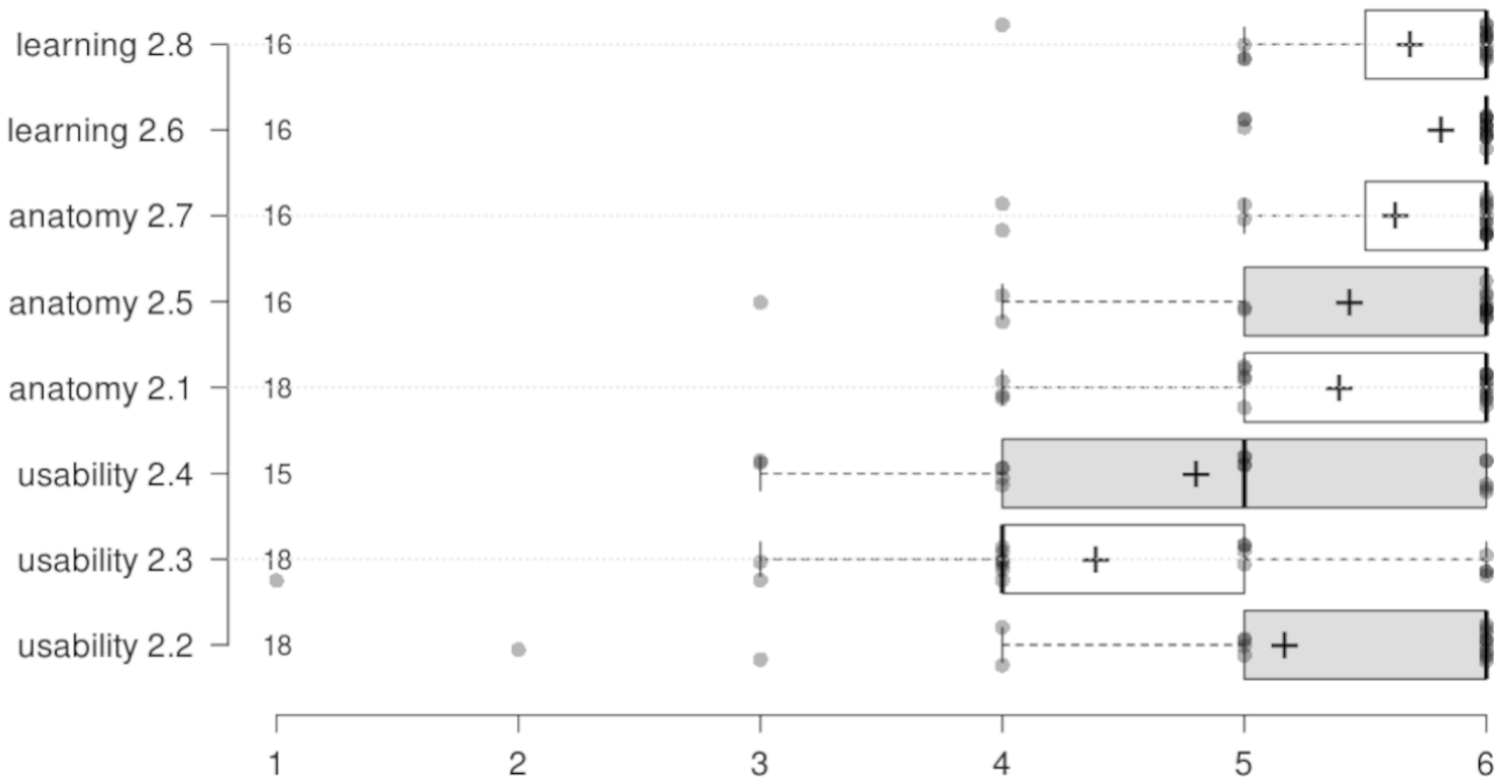
Boxplots of the responses collected from the group MR through the User Experience questionnaire on a 1-6 ordinal scale. For each item (i.e., row), the number indicates the progressive number of the item in the questionnaire (see Appendix Table 4 for the text), while the number besides the vertical axis indicates the number of respondents. Crosses indicate average scores

This indicates that holographic images helped students in better understanding the technical aspects and possible complications of SV-ASD transcatheter treatment. The results from the user experience questionnaire are reported in Fig. 6.

The analysis of the responses given by participants in the MR group via the user experience questionnaire shows that the students would recommend using immersive devices and holographic images: the Net Promoter Score is 72, with 78% of respondents being Promoters and 6% being Detractors. This is a very high value, since also slightly positive NPS values are usually associated with good services, due to the fact that only the highest scores (9 and 10) are associated to promoters. In particular, the highest scores were collected in regard to the Learning-related items (see Table 4 in the Appendix) and the Anatomy-related ones. The correlation between the average (normalized) scores of the Anatomy exploration section (see Table 4 in the Appendix) and the average (normalized) scores of the 4 treatment-related test items (see Table 3 in the Appendix) was found moderate (Pearson correlation coefficient .39 - although not significantly so, due to the low number of subjects involved, N=18): this means that the more the tool was considered useful and satisfactory by students in regard to anatomy exploration, the higher the level of knowledge acquired by students in regard to treatment, This finding suggests that such tools may have a beneficial impact on learning efficiency, accelerating the attainment of proficiency even for interventions that typically demand extensive manual practice sessions.

## Discussion

The findings of this study support the hypothesis that augmented reality technology and immersive devices are effective and engaging tools for undergraduate students learning heart anatomy. The main driver of higher teaching effectiveness appears to be the use of holographic models, either in a passive but dynamic setting like watching a video (group HV), or in an immersive and interactive setting (group MR). The global effect size of using holographic tools compared to traditional slides is medium (approximately 0.40), but the effect on individuals could be even greater.

All students were able to describe the anatomy of the taught defect (ASD-SV + PAPVR); however, only the students interacting with the holograms (either in the HV or the MR group) were able to answer the questions about planning, percutaneous treatment with stenting, and potential complications.

The transcatheter treatment of sinus venosus ASD associated with the anomalous pulmonary venous return (PAPVR) represents a new and interesting alternative that involves the implantation of a covered stent in the superior vena cava in order to close the defect and redirect the abnormal pulmonary veins in the left atrium. This type of treatment is not always feasible, depending on the type of anatomy of the pulmonary veins. Although these medical students (group HV and MR) were naive as regards the transcatheter treatment of CHD, they had, at the end of the lecture, a clear understanding of the principles and possible complications of the procedure. Holographic models helped them better understand the spatial position of the defect and its relationship with surrounding structures, including the risk of pulmonary vein compression by the stents.

The introduction of mixed reality holograms represents a recent and exciting advancement in educational technology. Students were able to actively learn content presented in their studies with the aid of the HoloLens, which increased their spatial understanding and overall engagement with the course material.

In general, the students agreed that the immersive devices helped them in understanding the anatomy of SVASD superior vena cava type and its transcatheter treatment (see the average scores for anatomy and learning items all above 5 in the 1-to-6 ordinal scale in Fig. 6). However, some students complained that the devices were difficult to use, especially at first attempt. As also mirrored by the scores from the UX questionnaire (see Fig. 6), students perceived this new way of using educational information as very inspiring,in line with the progressive shift in tertiary education from traditional lectures and tutorials to more self-paced, visual methods of learning [24]. This brings interesting challenges to education in medicine. Digital instructional tools, in the form of holographic models and immersive devices, present an opportunity to package content in an easy-to-comprehend way, enhancing the medical student learning experience. Researchers are testing how to enhance knowledge acquisition of health professional students [25]. Furthermore, the use of this technology enables improved educational practices as these 3D visualizations can be used on devices that are easily available [26, 27]. Based on the results of our user study, it is evident that the integration of mixed-reality devices in medical training holds tremendous potential in delivering interactive and immersive learning experiences. They allow students to simulate surgeries or diagnose patients, gaining practical skills and improving decision-making abilities without using cadavers or real patients. Moreover, these devices can improve accessibility and reduce costs, also providing a platform for remote learning. Additionally, the need for expensive physical equipment, such as cadavers or specialized training facilities, can be reduced via virtual simulations.

However, there are also challenges to using MR devices in medical training and education, including the need for simulations that closely mimic real-world situations. Ensuring accuracy and realism can be a difficult and time-consuming task, as it requires detailed knowledge of human anatomy and medical procedures. Another challenge is the potential for distraction and reduced focus in a virtual environment. Non-essential elements, such as background noise or other stimuli, can distract students and reduce their ability to concentrate and learn effectively. This can hinder the development of important skills.

Overall, the use of MR devices in medical training and education has the potential to provide significant benefits, but also comes with challenges that must be carefully considered and addressed.

## Limitations of the study

The main limitation of this study consists in the relatively small number of participants as it involved only a single class on a specific topic, thereby restricting the generalizability of the findings. Future work should entail a pre-class assessment to gauge the baseline (medical and technological) proficiency level of the students in each group, including their perception on the use of headsets and MR in a classroom setting. The final questionnaire could be improved by adding more items that more accurately assess the spatio-visual aspects of MR (such as labelling structures). Furthermore, a more in-depth, interview-based qualitative study could be devised, as to better explore students’ perceptions, expectations, hopes and worries concerning the use of MR during lessons. This is why we regard this study as exploratory and mainly aimed at estimating the effect size of this kind of educational intervention, which could inform the power analysis of further, more comprehensive and representative studies involving different classes and topics.

## Conclusions

In conclusion, this study demonstrates that the use of holographic heart models can improve the morphological understanding of complex congenital heart defects and their interventional planning and treatment. These models offer a unique way to visualize and analyze complex cadiological structures, while further research is needed to understand how best to integrate holographic technology into traditional teaching methods in medical education. The most interesting outcome of the study is the demonstration that the differences in performance between group MR and group HV are not statistically significant, suggesting no real measurable improvement in participant knowledge (MR vs HV) with the questionnaires employed. However, the use of MR displayed clear benefits in terms of user experience, demonstrating that immersive technology can effectively engage and inspire students. The enthusiasm and interest shown by the students in this study suggests that MR has the potential to revolutionize medical education, with more research needed to fully understand and capitalize on this new opportunity for teaching and training the upcoming generation of doctors.

## Appendix

**Table 2:** Items of the proficiency test. Correct answers are bolded except for question 5, as the correct answer consisted in entering the corresponding names for the structures shown in a heart diagram

ID	Domain
1. About the superior sinus venosus ASD prevalence	Knowledge
A They are rare defects with very low prevalence	
B **They represent about 10% of atrial septal defects**	
C They represent over 50% of atrial septal defects	
D They represent about 70% of atrial septal defects	
2. How would you define a superior sinus venosus ASD?	Anatomy
A A localized defect in the lower portion of the atrial septum (adjacent to the atrioventricular valves)	
B A confined defect in the region of the oval fossa	
C **A defect located in the postero-superior portion of the atrial septum near the outlet of the superior vena cava**	
D A stenosis of the superior vena cava at the outlet in the right atrium	
3. The superior sinus venosus ASDs are frequently associated with	Knowledge
A **Abnormal drainage of the right pulmonary veins**	
B Mitral valve cleft	
C Ventricular Septal Defect	
D Abnormal drainage of the left pulmonary veins	
4. What best describes the most common form of superior sinus venosus ASD?	Anatomy
A It is associated with the persistence of the left superior vena cava	
B Abnormal pulmonary veins always drain into the upper portion of the superior vena cava	
C The wall between the right inferior pulmonary vein and the Inferior Vena Cava is missing	
D **The wall between the right upper-middle pulmonary vein and the SVC is missing (pulmonary veins drain at the atrial cavity junction)**	
5. Enter the names of the following structures in the image below	Anatomy
A Superior sinus venosus ASD	
B Superior vena cava	
C Inferior vena cava	
D Inferior sinus venosus ASD

**Table 3:** Items of the proficiency test (cont.)

ID	Domain
6. The superior sinus venosus ASD can be repaired	Treatment
A Only by surgery	
B It cannot be corrected	
C **Surgery or transcatheter treatment**	
D Only with transcatheter treatment	
7. Transcatheter correction is possible with	Treatment
A **Covered stent implantation**	
B Double disk device implantation	
C Uncovered stent implantation	
D Transcatheter treatment is not possible	
8. Transcatheter correction is possible with	Treatment
A **The abnormal drainage is at the cavo-atrial junction**	
B Always possible	
C The abnormal drainage of the pulmonary veins is in the upper portion of the superior vena cava	
D There is an anchor point in superior vena cava	
9. During the catheterization, balloon catheters are used to	Treatment
A Dilate the superior vena cava	
B Dilate the pulmonary veins	
C Measure the diameters	
D **Simulate the implantation of the stent in the superior vena cava**	
10. What is the most severe complication of transcatheter treatment with a covered stent?	Treatment
A Occlusion of the superior vena cava	
B Interference with the coronary sinus	
C Interference with the right coronary artery	
D **Stenosis of the right pulmonary veins**

**Table 4:** Items of the UX questionnaire

ID	Item	Domain
1	“On a scale from 0 to 10, what is the likelihood that you would recommend to a colleague or friend to attend a lecture conducted via holographic images and immersive devices?”	Recommendability
2	On a scale from 1 (strongly disagree) to 6 (strongly agree) (+ one “don’t know” option) indicate your degree of agreement with the following statements	User Experience (incl. Usability, Anatomy exploration, Learning experience)
2.2	I think it would be easy for me to become proficient in using the immersive device	Usability
2.3	I can use the immersive device easily and without special help	Usability
2.4	I was able to do “air tapping” and other gestures to interact with the holographic images	Usability
2.1	It was easy to explore the anatomy and observe the interatrial sinus venous defect with the immersive device	Anatomy exploration
2.5	The holographic images enhanced and strengthened my understanding of the anatomical defect that was the subject of the lecture	Anatomy exploration
2.7	The holographic images can represent the cardiac anatomy adequately	Anatomy exploration
2.6	I believe that holographic images are useful in medical learning and training	Learning Experience
2.8	These immersive devices are useful in my course of study to enhance the enjoyment of lectures and the understanding of the topics covered	Learning Experience

## References

[1] Giovanni Biglino, Despina Koniordou, Marisa Gasparini, Claudio Capelli, Lindsay-Kay Leaver, Sachin Khambadkone, Silvia Schievano, Andrew M Taylor, and Jo Wray. Piloting the use of patient-specific cardiac models as a novel tool to facilitate communication during cinical consultations. *Pediatric Cardiology*, 38(4):813–818, 2017. 10.1007/s00246-017-1586-9 PMC5388703 28214968

[2] Lau Ivan, Sun Zhonghua (2018). Three-dimensional printing in congenital heart disease: A systematic review. Journal of medical radiation sciences.

[3] Lee Hyangsook (2013). 3d holographic technology and its educational potential. TechTrends.

[4] Jan-Philipp Stauffert, Florian Niebling, and Marc Erich Latoschik. Latency and cybersickness: Impact, causes, and measures. a review. *Frontiers in Virtual Reality*, 1:582204, 2020.

[5] Moro Christian, Phelps Charlotte, Jones Dominique, Stromberga Zane (2020). Using holograms to enhance learning in health sciences and medicine. Medical Science Educator.

[6] Prabha Susy Mathew and Anitha S Pillai. Role of immersive (xr) technologies in improving healthcare competencies: a review. *Virtual and Augmented Reality in Education, Art, and Museums*, pages 23–46, 2020.

[7] Min-Hsien Lee and Chin-Chung Tsai. Exploring teachers’ perceived self efficacy and technological pedagogical content knowledge with respect to educational use of the world wide web. *Instructional Science*, 38(1):1–21, 2010.

[8] Nico Rutten, Wouter R Van Joolingen, and Jan T Van Der Veen. The learning effects of computer simulations in science education. *Computers & education*, 58(1):136–153, 2012.

[9] Miller Michael (2016). Use of computer-aided holographic models improves performance in a cadaver dissection-based course in gross anatomy. Clinical Anatomy.

[10] Santiago González Izard, Juan A Juanes Méndez, Pablo Ruisoto Palomera, and Francisco J García-Peñalvo. Applications of virtual and augmented reality in biomedical imaging. *Journal of medical systems*, 43(4):1–5, 2019. 10.1007/s10916-019-1239-z 30874965

[11] Zhonglin Qu, Chng Wei Lau, Simeon J Simoff, Paul J Kennedy, Quang Vinh Nguyen, and Daniel R Catchpoole. Review of innovative immersive technologies for healthcare applications. *Innovations in Digital Health, Diagnostics, and Biomarkers*, 2(2022):27–39, 2022.

[12] Li Qiming, Huang Chen, Lv Shengqing, Li Zeyu, Chen Yimin, Ma Lizhuang (2017). An human-computer interactive augmented reality system for coronary artery diagnosis planning and training. Journal of medical systems.

[13] Bork Felix, Stratmann Leonard, Enssle Stefan, Eck Ulrich, Navab Nassir, Waschke Jens, Kugelmann Daniela (2019). The benefits of an augmented reality magic mirror system for integrated radiology teaching in gross anatomy. Anatomical sciences education.

[14] Hackett Matthew, Proctor Michael (2018). The effect of autostereoscopic holograms on anatomical knowledge: a randomised trial. Medical education.

[15] Stefano Triberti, Francesco Petrella, Alessandra Gorini, Omar Pappalardo, Valeria Sebri, Lucrezia Savioni, Alberto Redaelli, and Gabriella Pravettoni. Augmenting surgery: medical students’ assessment and ergonomics of 3d holograms vs. ct scans for pre-operative planning. *EAI Endorsed Transactions on Pervasive Health and Technology*, 7(25):e5–e5, 2021.

[16] Yuwei Ji, Xiangjun Zhang, Hanze Tang, Hao Luo, Shengwei Zhao, Zhaowen Qiu, Qinghua Zhang, Kun Wang, and Liwei Diao. Education platform of congenital heart disease based on mixed reality technology. In *International Conference of Pioneering Computer Scientists, Engineers and Educators*, pages 313–334. Springer, 2019.

[17] Zane Stromberga, Charlotte Phelps, Jessica Smith, and Christian Moro. Teaching with disruptive technology: the use of augmented, virtual, and mixed reality (hololens) for disease education. In *Biomedical Visualisation*, pages 147–162. Springer, 2021. 10.1007/978-3-030-61125-5_8 33945136

[18] Moro Christian, Phelps Charlotte, Redmond Petrea, Stromberga Zane (2021). Hololens and mobile augmented reality in medical and health science education: A randomised controlled trial. British Journal of Educational Technology.

[19] chen chen, Lei Zhang, Tony Luczak, Eboni Smith, Reuben F Burch, et al. Using microsoft hololens to improve memory recall in anatomy and physiology: A pilot study to examine the efficacy of using augmented reality in education. *Journal of Educational Technology Development and Exchange (JETDE)*, 12(1):2, 2019.

[20] Elizabeth A Duncan-Vaidya and Erica L Stevenson. The effectiveness of an augmented reality head-mounted display in learning skull anatomy at a community college. *Anatomical Sciences Education*, 14(2):221–231, 2021. 10.1002/ase.1998 32583577

[21] DF Hamilton, Judith V Lane, P Gaston, JT Patton, DJ Macdonald, AHRW Simpson, and CR Howie. Assessing treatment outcomes using a single question: the net promoter score. *The bone & joint journal*, 96(5):622–628, 2014. 10.1302/0301-620X.96B5.32434 24788496

[22] Cabitza Federico, Locoro Angela (2017). Questionnaires in the design and evaluation of community-oriented technologies. International journal of web based communities.

[23] Lakens Daniël (2013). Calculating and reporting effect sizes to facilitate cumulative science: a practical primer for t-tests and anovas. Frontiers in psychology.

[24] Christian Moro and Michelle McLean. Supporting students’ transition to university and problem-based learning. *Medical Science Educator*, 27(2):353–361, 2017.

[25] Birt James, Stromberga Zane, Cowling Michael, Moro Christian (2018). Mobile mixed reality for experiential learning and simulation in medical and health sciences education. Information.

[26] Christian Moro, Zane Stromberga, and James Birt. Technology considerations in health professions and clinical education. *Clinical education for the health professions: Theory and practice*, pages 1–22, 2020.

[27] Christian Moro and Sue Gregory. Utilising anatomical and physiological visualisations to enhance the face-to-face student learning experience in biomedical sciences and medicine. In *Biomedical Visualisation*, pages 41–48. Springer, 2019. 10.1007/978-3-030-19385-0_3 31338776

